# Investigating pulmonary inflammation and injury after progressive systemic inflammation in preterm fetal sheep

**DOI:** 10.3389/fphys.2025.1542613

**Published:** 2025-05-15

**Authors:** E. G. Vandenberg, S. B. Kelly, V. A. Zahra, H. Lu, A. Thiel, S. B. Hooper, R. Galinsky, G. R. Polglase

**Affiliations:** ^1^ The Ritchie Centre, Hudson Institute of Medical Research, Melbourne, VIC, Australia; ^2^ Department of Paediatrics, Monash University, Melbourne, VIC, Australia; ^3^ Department of Obstetrics and Gynaecology, Monash University, Melbourne, VIC, Australia

**Keywords:** chorioamnionitis, infection, lung, sepsis, preterm birth

## Abstract

**Introduction:**

Preterm birth and intrauterine inflammation are commonly associated with lung inflammation and remodeling. We developed a fetal inflammatory response model using increasing doses of intravenous lipopolysaccharide (LPS) to cause systemic inflammation and injury. However, the effects of an increasing systemic inflammatory response on fetal lung inflammation and injury are not known. We aimed to investigate the effect of repeated increasing doses of intravenous LPS on pulmonary inflammation and injury in preterm fetal sheep.

**Methods:**

Fetal sheep at 124 days of gestation (term ∼148 days) underwent surgical instrumentation. At 129 days of gestation, fetal sheep were randomized to saline control (n = 8) or repeated LPS infusions (300 ng/24 h then doubled every 24 h for 2 days; n = 8). Four days after LPS/saline infusions commenced, fetal lungs were collected for histological and molecular analysis of markers of pulmonary inflammation and injury.

**Results:**

Repeated increasing doses of intravenous LPS decreased arterial pH, PaO_2_, SaO_2_ and increased lactate and PaCO_2_ compared to controls. LPS infusions caused a decrease in mRNA expression of pro-inflammatory cytokines *IL1B* (p = 0.030) and *IL6* (p = 0.034) and an increase in *IL18* (p < 0.0001). LPS exposure did not alter histological assessment of airway structure, elastin or collagen abundance, inflammatory cell infiltration or cell death compared to controls.

**Conclusion:**

Intravenous administration of LPS did not cause fetal lung inflammation and injury assessed 4 days after LPS infusions commenced. Direct exposure to endotoxins within the lungs may be necessary to induce inflammation and injury in the fetal lungs.

## 1 Introduction

Intrauterine infection/inflammation is the most common cause of preterm birth. It is commonly associated with lung inflammation, injury and remodeling and increases the risk of chronic lung injury such as bronchopulmonary dysplasia (BPD) (Moss et al., 2002; Galinsky et al., 2013a; Watterberg et al., 1996).

Exposure to inflammation *in utero* often evokes a fetal inflammatory response, termed fetal inflammatory response syndrome (FIRS), which is categorized by elevated pro-inflammatory cytokines and subsequent multi-organ injury, including pulmonary inflammation and lung tissue remodeling (Galinsky et al., 2013a; Kramer et al., 2010). Within the lungs, bacteria are recognized by toll-like receptors (TLRs), initiating an innate and adaptive immune response, incorporating recruitment of T-lymphocytes, increases in white blood cells (monocytes, neutrophils and lymphocytes) and the downstream activation of pro-inflammatory cytokines which impairs alveolar septation (Kakkera et al., 2005; Willet et al., 2002; Cheah et al., 2009; Kallapur et al., 2011; Kramer et al., 2005) resulting in a lung with few, larger alveoli and a reduced surface area for gas exchange. Furthermore, the FIRS causes thickening of the arterioles which increases pulmonary vascular resistance (Kallapur et al., 2004). Together, the remodeling of the pulmonary vasculature and airways, caused by intrauterine inflammation, impairs gas exchange and is associated with lung injury characteristic of bronchopulmonary dysplasia (Kallapur et al., 2004; Stocker, 1986).

Lipopolysaccharide (LPS) is widely used to model intrauterine inflammation and fetal/newborn organ injury. Studies conducted in preterm fetal sheep have shown that intraamniotic administration of LPS increases markers of systemic and pulmonary inflammation (Kramer et al., 2001; Kallapur et al., 2001), resulting in lung remodeling, such as increased collagen and elastin deposition, fewer and larger alveoli and a thicker blood gas barrier, which together reduces the ability for gas exchange (Moss et al., 2002; Kallapur et al., 2004; Willet et al., 2000; Smith et al., 2010). Studies in fetal sheep exposed to intra-amniotic lipopolysaccharide showed that pulmonary inflammation and tissue remodeling does not occur if the lungs are isolated from the amniotic fluid (Moss et al., 2002). Whilst intra-amniotic LPS administration produces a profound FIRS within the lungs, it does not mimic the paradigm of the progressive increase in systemic inflammation which often occurs during an evolving intrauterine infection. We developed a model of progressive systemic inflammation whereby preterm sheep fetuses are exposed to daily increasing doses of intravenous LPS (Kelly et al., 2021). This resulted in systemic and neural inflammatory response, and brain pathology (Kelly et al., 2021). However, it is unknown whether progressive systemic inflammation, induced by increases doses of intravenous LPS causes lung inflammation and injury. In this study, we aimed to determine the role of progressive systemic inflammation, induced by increases doses of intravenous LPS on markers of lung inflammation and injury in preterm fetal sheep. We hypothesized that exposure to increasing intravenous infusions of LPS will cause lung inflammation and parenchymal remodeling.

## 2 Materials and methods

All procedures were approved by Monash Medical Centre “A” Animal Ethics Committee and were conducted in accordance with the National Health and Medical Research Council (NHMRC) *Australian code for the care and use of animals for scientific purposes*. The animals included for analysis in this study are a subset of animals previously published (Kelly et al., 2021; Kelly et al., 2023; Stojanovska et al., 2022). Experiments are reported in accordance with the ARRIVE guidelines (Percie du Sert et al., 2020).

### 2.1 Fetal instrumentation

Under aseptic conditions, pregnant Border-Leicester ewes bearing singleton or twin fetuses underwent surgery at 124 ± 1 days of gestation, as described in detail previously (Kelly et al., 2021). Briefly, anesthesia was induced by i.v injection of sodium thiopentone (20 mL) and maintained using 2%–3% isoflurane in oxygen (Bomac Animal Health, New South Wales, Australia). Ewes received prophylactic antibiotics (ampicillin, 1 g i.v; Austrapen, Lennon Healthcare, St. Leonards, New South Wales, Australia, and engemycin, 500 mg i.v; Schering-Plough, Upper Hutt, New Zealand) immediately before surgery.

A midline maternal laparotomy was performed, the fetal sheep was exteriorized, and polyvinyl catheters were inserted into the right brachiocephalic artery, axillary vein and amniotic cavity. In the case of a twin pregnancy, only one fetus was operated on. The fetus was returned to the amniotic cavity in its original orientation and all fetal leads were exteriorized through the maternal flank. A catheter was inserted into the maternal jugular vein for administration of post-operative antibiotics. Ewes and fetal sheep were administered prophylactic antibiotics for 3 days post-surgery. 24 h between antibiotic administration and the start of the experiment prevented attenuation of the inflammatory response. At the completion of surgery, post-operative analgesia was provided to the ewe for 3 days via a transdermal fentanyl patch (75 μg/h patch Janssen Cilag, North Ryde, NSW, Australia), placed on the left hind leg.

Ewes were housed together in separate mobile pen in a temperature (20°C ± 1°C) and humidity (50% ± 10%) controlled room with a 12-h light-dark cycle with *ad libitum* access to food and water. Ewes and fetuses were allowed four to 5 days to recover after surgery before commencement of experiments.

### 2.2 Experimental protocol

At 129 ± 1days gestation, fetal sheep were randomly allocated to either control (saline, n = 8) or LPS (*Escherichia coli*, 055: B5, MilliporeSigma, MO, USA, n = 8). LPS-exposed fetuses received 300 ng, 600 ng and 1,200 ng infusions of intravenous LPS diluted in 2 mL of saline at an infusion rate of 1 mL/min at 0 h, 24 h and 48 h, respectively. Controls received an equivalent volume of i.v saline at the same timepoints and infusion rate. This experimental protocol aimed to model the progressive systemic inflammation that occurs during FIRS. Daily arterial blood samples at 0900 h (baseline) and two and 6 hours after saline/LPS infusions were collected from the right brachiocephalic artery for analysis of blood gas composition (ABL 90 Flex Plus analyzer, Radiometer, Brønshøj, Denmark).

At the end of the study (133 days gestation, 48 h after the last LPS infusion), the ewe and fetus were euthanized by pentobarbital sodium (100 mg/kg, Lethobarb, Virbac Pty, New South Wales, Australia), administered via the maternal jugular vein catheter.

### 2.3 Lung collection and processing

At post-mortem, the fetus was exteriorized from the uterus and the sex and body weight recorded. Segments of the left lung were snap frozen in liquid nitrogen for molecular analysis. The right lung was perfusion-fixed at 20 cm H_2_O with 10% formalin, separated into upper, middle and lower lobes and fixed in Zamboni’s fixative solution. 1.5 cm^2^ sections were processed and embedded in paraffin wax blocks. The blocks were cut using a microtome (Jung Biocut 2035 Geprufte Sicherhiet Germany) at a thickness of 5 microns, placed in a 38°C water bath and mounted on Superfrost microscope slides (Superfrost Plus, Thermo Scientific, Germany) for histological analysis.

### 2.4 Lung histology

Sections of the right upper lobe were incubated at 60°C for 60 min, dewaxed with xylene and rehydrated with decreasing concentrations of ethanol. Sections were stained with Hematoxylin and eosin for analysis of gross lung pathology, Hart’s resorcin-fuchsin for elastin analysis and picrosirius red for collagen analysis, as described previously (Polglase et al., 2017).

### 2.5 Lung inflammatory cell infiltration

Citric acid buffer (pH = 6) was used for heat-mediated antigen retrieval using a microwave for 16 min. Endogenous peroxide blocking was performed by incubating slides in 0.3% H_2_O_2_ for 20 min. Sections were blocked from non-specific antibody binding by incubation in a solution of 10% normal goat serum (10% Normal Goat Serum/2% Bovine Serum Albumin/Tris buffered saline). Sections were labelled with primary antibody, Mouse anti Sheep CD45 (Mouse anti Sheep CD45, 1:200 dilution; Bio Rad, Hercules, United States, Cat. # MCA2220GA), overnight at 4°C. Slides were incubated with secondary antibody, Goat anti Mouse IgG (Goat anti Mouse IgG, 1:400 dilution: Vector Laboratories, CA, United States Cat. # BA-9200–1.5; 200 µl/slide), for 60 min. Slides were then incubated with Avidin-Biotin Complex (ABC, Thermo Fisher Scientific, MA, United States; 200 µl/slide) for 45 min, followed by 3,3′-Diaminobenzidine complex (DAB, Thermo Fisher Scientific, MA, United States; 200 µl/slide) for 5 min. Sections were counterstained with Hematoxylin, dehydrated in xylene and cover slipped.

### 2.6 Lung cell death

Terminal deoxynucleotidyl transferase dUTP nick end labelling (TUNEL) was used to identify cells undergoing *in situ* apoptosis using the ApopTag Peroxidase Kit as per manufacturer’s instructions (Millipore S7100; CA, USA). Proteinase K was applied to sections to perform antigen retrieval. Endogenous peroxide quenching was performed by incubating slides in 0.3% H_2_O_2_ in phosphate buffered saline (PBS) for 5 min. Equilibrium buffer was then applied to sections for 5 min, followed by terminal deoxynucleotidyl transferase enzyme for 60 min. Slides were incubated in working stop/wash buffer for 10 min then incubated in anti-digoxigenin conjugate for 30 min before being incubated with diaminobenzidine peroxidase substrate. DAB was applied to sections for 4 min. PBS was used to stop the reaction. Slides were counterstained with Hematoxylin, dehydrated in xylene and cover slipped.

### 2.7 Quantification of lung histology

Slides were imaged using light microscopy (Olympus BX53 Microscope, Tokyo, Japan) at ×40 magnification (CD45, TUNEL, Elastin, Collagen) or ×100 magnification (H&E). Ten random, non-overlapping fields of view (excluding major airways or blood vessels) were captured of each section (Olympus DP27 Color Camera, Tokyo, Japan). All sections were assessed by a single observer blinded to the experimental group. H&E staining was used to assess relative lung tissue to airspace volumes and developing secondary septal crest abundance using a validated multipurpose test grid overlaid on each image (Hsia et al., 2010). Briefly, a coherent test grid containing 21 short test lines of known length, each with 2 ends with test points, was overlaid on the image. Manual counting was performed to determine the number of test points falling on parenchymal lung tissue and alveolar airspace. This was used to determine percentage of tissue and airspace per field of view. The number of intersections crossing the tissue-air interface was counted to determine mean linear intercept and alveolar wall thickness, which were calculated as per the guidelines of quantitative lung structure assessment (Hsia et al., 2010). Elastin and collagen density were assessed using the threshold function on Fiji ImageJ processing software and quantified relative to tissue area. Hart’s resorcin-fuchsin was used to assess developing secondary septal crests abundance. Briefly, a grid overlay with 144 dots (12 × 12 points, 25 μm apart) was placed over each image using Fiji ImageJ processing software. Points falling on secondary septal crests, distinguished by elastin deposition at the tips, were counted. The number of secondary septal crests was expressed as a percentage per field of view and a percentage relative to tissue area. Manual counting of positively stained CD45 and TUNEL cells was performed. Positive CD45 cells were expressed as a percentage of total cell number, which was obtained using a Fiji ImageJ macro while TUNEL positive cells were expressed as number of positive cells per field of view.

### 2.8 Lung mRNA analysis

Real-time quantitative polymerase chain reaction (RT-qPCR) was undertaken to quantify mRNA expression of inflammatory cytokines (*IL1A, IL1B, IL6, IL8, IL18* and *NLRP3*) and surfactant proteins (*SP-A, SP-B, SP-C* and *SP-D*) in lung tissue.

Total RNA was extracted from snap-frozen lung tissue (∼600 mg) using the RNeasy Maxi RNA extraction kit (Qiagen, Australia) and reverse-transcribed into complementary DNA (cDNA) as per protocols of the SuperScript® III First-Strand Synthesis System for RT-PCR kit (Invitrogen, CA, USA). Gel electrophoresis was used to validate the quality of extracted RNA and check for DNA contamination. mRNA expression of the genes of interest were measured by qRT-PCR using a Thermo Fisher Quantstudio 6 Real Time PCR machine. Each sample was performed in triplicate and analyzed at the point at which the amplification plot crossed the threshold value (Ct). mRNA expression was normalized to the housekeeping gene 18S by subtracting the Ct from the Ct of the gene of interest (ΔCt). 18s was chosen as the housekeeping gene as its values were not altered by LPS in this study, as well as previous studies by our group. The mRNA levels for each sample were then normalized using the equation 2-ΔΔCt (Bustin et al., 2009) and the results were expressed relative to the mean mRNA expression levels of the control group.

### 2.9 Data analysis and statistics

Data was analyzed by GraphPad Prism (Prism v10, GraphPad Software, San Diego, CA). Fetal characteristics, molecular and immunohistochemical data were compared using a Student’s t-test (unpaired) and presented as means ± standard error of the mean (SEM). Blood gas variables were analyzed using a two-way repeated measures ANOVA with treatment and time as independent factors and presented as means ± standard deviation (SD). A post-hoc Sidak’s multiple comparisons test was used to determine time x treatment interactions. Statistical significance was accepted when p < 0.05.

## 3 Results

### 3.1 Fetal characteristics

Fetal sheep characteristics are presented in Table 1. No significant difference in any of these parameters was found between groups.

**TABLE 1:** Fetal characteristics.

Characteristics	Control	LPS	p-value
Number	8	8	—
Sex	2 Females, 6 Males	1 Female, 7 Males	—
Singleton/Twin	6 Singletons, 2 Twins	5 Singleton, 3 Twins	—
Body Weight (kg)	4.67 ± 0.21	4.63 ± 0.19	0.903
Lung Weight (g)	186.9 ± 8.06	173.6 ± 18.58	0.520
Lung corrected for body weight (g/kg)	40.40 ± 2.02	37.26 ± 3.12	0.412
Lung protein concentration (mg/g)	22.74 ± 2.036	23.88 ± 1.564	0.717
Lung protein content (mg/kg)	916.9 ± 68.98	880.2 ± 70.16	0.660

Data presented as mean ± SEM.

### 3.2 Fetal blood gas analysis

**FIGURE 1:**
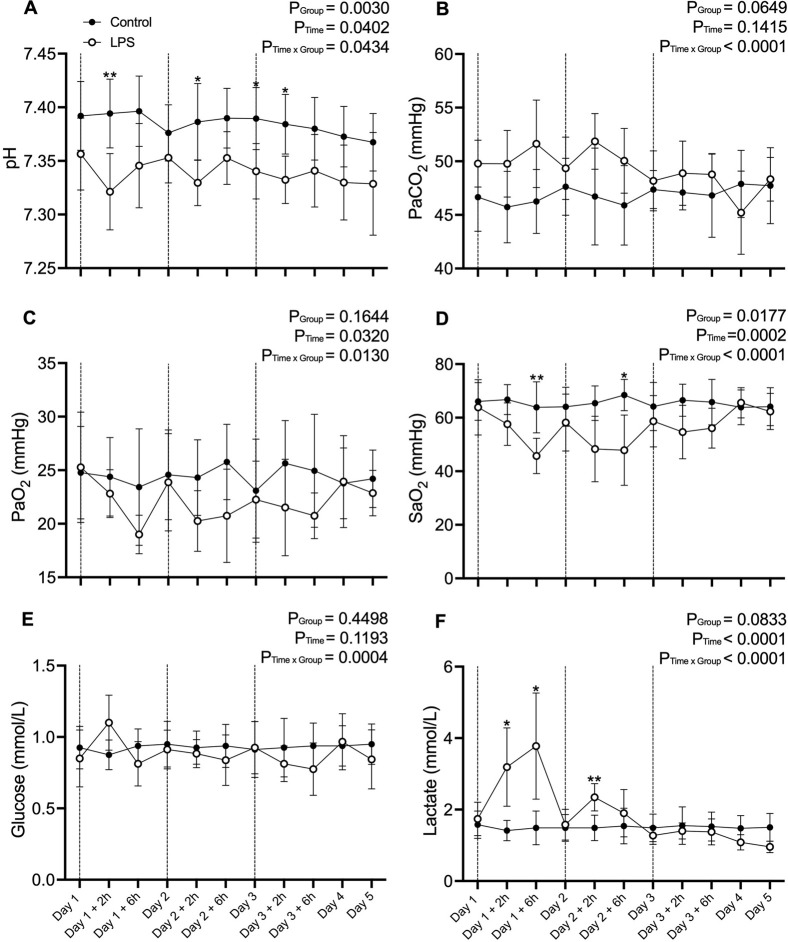
Fetal blood gas data. **(A)** pH, **(B)** Partial pressure of arterial (Pa) carbon dioxide (CO_2_), **(C)** Pa oxygen (O_2_), **(D)** Saturation of arterial oxygen (SaO_2_), **(E)** glucose and **(F)** lactate in control (black circles; n = 8) and LPS (open circles; n = 8) groups across the five experimental days. Values are temperature corrected. Dotted lines represent LPS administration. Data are mean ± SD. *p < 0.05, **p < 0.01.

Fetal blood gas variables are presented in Figure 1. pH was lower in the LPS group compared to control 2 h after LPS infusions on day one and two of the experiment (p < 0.05; Day 1 + 2 h and Day 2 + 2 h and from baseline to 2 h after LPS administration on day three (p < 0.05; Day 3 – Day 3 + 2 h). SaO_2_ was lower in the LPS group compared to controls 6 h after LPS was administered on days 1 and 2 of the experiment (p < 0.05; Day 1 + 2 h and Day 2 + 6 h). Lactate was higher in the LPS group compared to the control group on day one of the experiment (p < 0.05; Day 1 + 2 h–Day 1 + 6 h) and 2 hours after LPS administration on day two (p < 0.05; Day 2 + 2 h). There were no differences in PaCO_2_, PaO_2_ or glucose between groups throughout the study period.

### 3.3 Lung morphology

#### 3.3.1 Gross lung morphology

**FIGURE 2:**
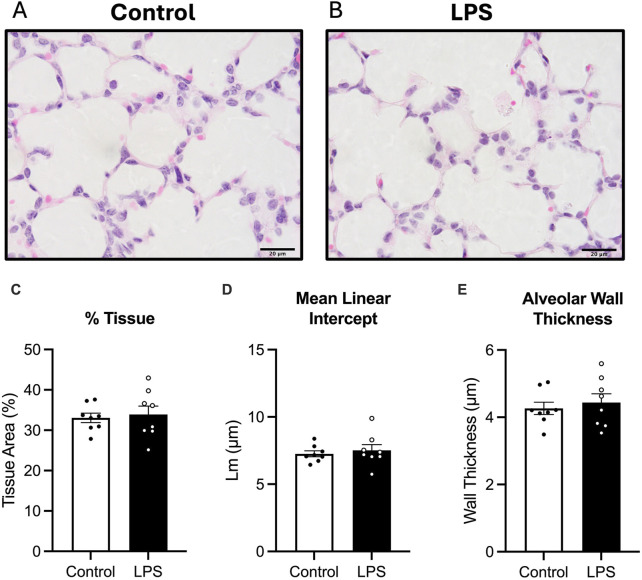
Analysis of haematoxylin and eosin, (H&E) stain. Representative photomicrographs of H&E stain **(A,B)**, Tissue to airspace ratio **(C)**, mean linear intercept **(D)** and alveolar wall thickness **(E)** for control (white bars: n = 8) and LPS (black bars; n = 8). Data are mean ± SEM with individual animals shown as circles. Scale bar = 20 µm.

Average tissue to airspace ratio, mean linear intercept and alveolar wall thickness was not different between the LPS and control groups (Figure 2).

#### 3.3.2 Elastin and collagen analysis

**FIGURE 3:**
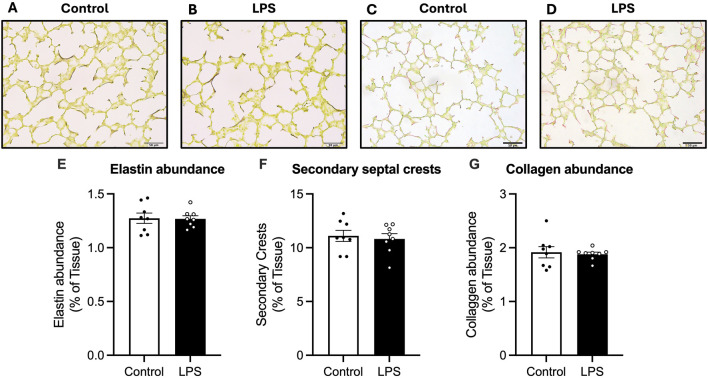
Analysis of Hart’s resorcin-fucshin and picrosirius red staining in the lung parenchyma. Representative photomicrographs of Hart’s **(A,B)** and picrosirius red stain **(C,D)**. Elastin abundance as a percentage of tissue **(E)**, secondary septal crests **(F)** and collagen abundance as a percentage of total tissue abundance **(G)** for control (white bars: n = 8) and LPS (black bars; n = 8). Data are mean ± SEM with individual animals shown as circles. Scale bar = 50 µm.

Total elastin abundance, number of secondary crests and number of secondary septal crests expressed as a percentage of total tissue in the lungs was not different between control and LPS fetuses (Figure 3).

Collagen abundance was not different between control and LPS groups.

### 3.4 Immunohistochemistry

#### 3.4.1 Cellularity and inflammatory cell infiltration

**FIGURE 4:**
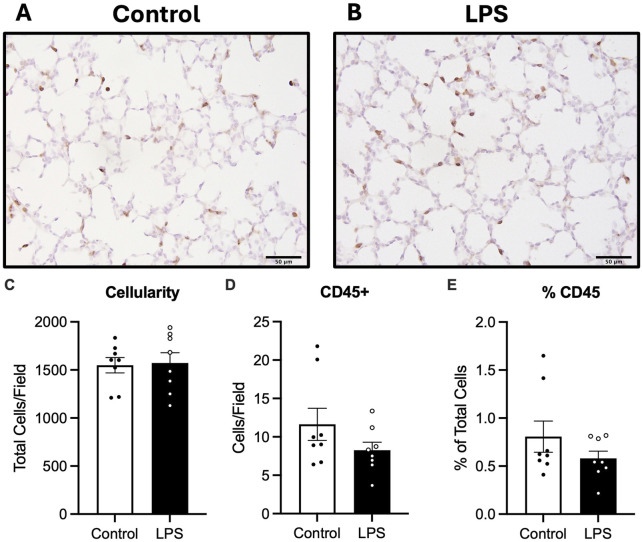
Analysis of CD45 staining in lung tissue. Representative photomicrographs of CD45 stain **(A,B)**, total cell count as a measure of cellularity **(C)** number of CD45 positive cells **(D)** and number of CD45 positive cells as a ratio of total cells **(E)** for control (n = 8) and LPS (n = 8). Data are mean ± SEM. Scale bar = 50 µm.

Cellularity of the lung parenchyma was determined by counting the total number of cells per field of view. No differences in total number of cells within the lung tissue were observed between groups (Figure 4). The total number of CD45 positive cells and the proportion of CD45 positive cells relative to total cells in lung tissue were not different between the control and LPS groups (Figure 4).

#### 3.4.2 Cell death

**FIGURE 5:**
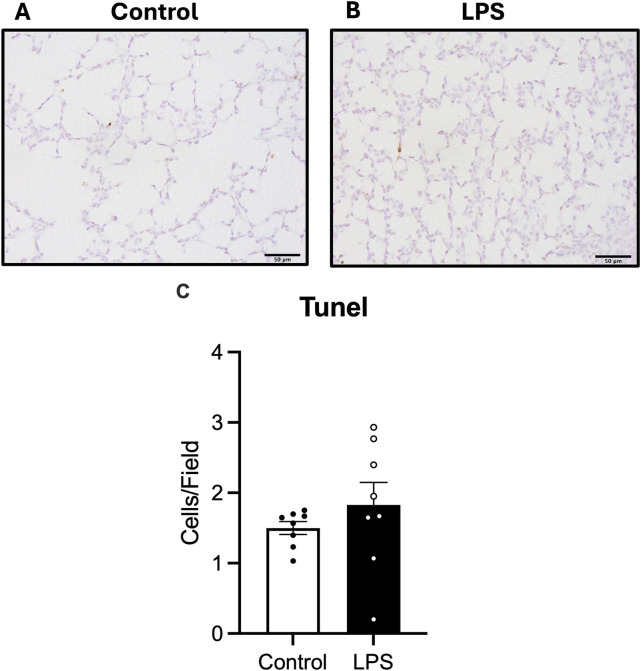
Analysis of TUNEL staining in lung tissue. Representative photomicrographs of TUNEL stain **(A,B)** and number of TUNEL positive cells **(C)** in control (white bars: n = 8) and LPS (black bars; n = 8). Data are mean ± SEM with individual animals shown as circles. Scale bar = 50 µm.

The number of cells undergoing apoptotic cell death in the lung parenchyma was assessed by quantifying numbers of TUNEL positive cells. There was no difference in numbers of TUNEL positive cells between the control and LPS groups (Figure 5).

### 3.5 Inflammation and surfactant protein gene expression

mRNA levels of *IL1B* and *IL6* were decreased in fetuses exposed to LPS compared to controls (p = 0.030 and p = 0.034 respectively). *IL18* mRNA expression was increased in the LPS group compared to controls (p < 0.0001). *IL8* and *NLRP3* mRNA expression was not different between groups. The levels of *IL1A* were below the detectable threshold in both groups (data not shown).

**FIGURE 6:**
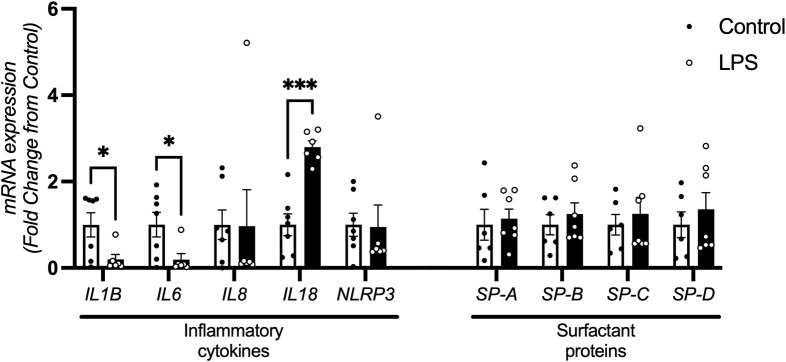
Inflammatory cytokine and surfactant protein mRNA expression in the lung. Lung mRNA levels of cytokines *IL1B*, *IL6*, *IL8*, *IL18* and *NLRP3* control (n = 7) and LPS (n = 6) and surfactant proteins *SP-A, SP-B, SP-C* and *SP-D* for control (n = 7) and LPS (n = 7) (fold change from control). Data are mean + SEM. *P < 0.05, ***P < 0.0001.

mRNA expression of surfactant proteins *SP-A, SP-B, SP-C* and *SP-D* were not different between the groups (Figure 6).

## 4 Discussion

We investigated markers of lung inflammation and injury after progressive systemic inflammation caused by increasing doses of intravenous LPS in preterm fetal sheep. Despite inducing progressive systemic inflammation, we did not find evidence of lung inflammation or changes to lung structure in fetuses exposed to intravenous LPS. Our findings suggest that systemic inflammation induced by repeated increasing doses of intravenous LPS to the fetus does not affect markers of pulmonary inflammation and injury in preterm fetal sheep.

Intrauterine inflammation/infection is associated with the onset of the fetal inflammatory response syndrome (Rocha, 2013; Gomez et al., 1998). Intrauterine inflammation commonly manifests from an ascending bacterial infection (Goldenberg et al., 2000), whereby microbes migrate from the vagina through the cervix to the choriodecidual space (Galinsky et al., 2013a; Goldenberg et al., 2000; Romero et al., 2001). Once inside the choriodecidual space, the microbes can penetrate the fetal membranes and invade the amniotic space (Galinsky et al., 2013a; Espinoza et al., 2003). The bacteria gain access to the fetus through fetal breathing movements or fetal swallowing. The pathogen must come into direct contact with the fetal lungs or gut to elicit an inflammatory response, as studies have shown that isolating the lungs and gut from the amniotic cavity containing endotoxins prevents a localized inflammatory response from occurring (Moss et al., 2002; Wolfs et al., 2014).

Gram-negative bacteria are one of the most common pathogens involved in intrauterine infection and inflammation (Sherman et al., 1997). Many studies have attempted to model the effects of gram negative intrauterine infection and inflammation on lung inflammation and injury by inducing intraamniotic inflammation through direct injection of lipopolysaccharide into the amniotic cavity (Willet et al., 2002; Kramer et al., 2001; Kallapur et al., 2001). However, this model fails to replicate the progressive increase in systemic inflammation that occurs during FIRS. We established a model of escalating intravenous LPS infusions in order to study the effect of the progressive systemic inflammatory response that occurs during an evolving intrauterine infection, on multi-organ inflammation and injury. We recently showed that intravenous LPS resulted in a profound systemic and cerebral inflammatory response in the same model used in our study (Kelly et al., 2021; Kelly et al., 2023). LPS infusions increased levels of systemic cytokines, including IL-1β, IL-6, IL-10 and TNF. Additionally, LPS caused a localized inflammatory response within the brain, demonstrated by EEG suppression and increased IL-1β immunoreactivity and number of caspase 3+ cells and microglia. In contrast, our study found reduced IL-1β and IL-6 mRNA expression within the lung, and no change in cellularity or immune cell infiltration suggesting a minimal inflammatory response by the lungs. Based on the profound effects of this model on systemic and cerebral inflammation, we wanted to know what effect systemic inflammation *per se* has on lung inflammation and development.

In previous studies investigating the consequences of intrauterine inflammation on lung structure and function, intra-amniotic injection of LPS in pregnant sheep caused activation of the FIRS which disrupted the normal progression of lung development. Indeed, these studies reported altered elastin and collagen content and distribution, altered surfactant production and remodeling of the lung vasculature and parenchyma resulting in fewer but larger alveoli, consistent with that observed in preterm infants with BPD (Willet et al., 2002; Kallapur et al., 2004; Rocha, 2013; Gotsch et al., 2007; Papagianis et al., 2019; Kallapur et al., 2005). Additionally, direct exposure to IL-1α or IL-1β via intraamniotic injection has shown to elicit a similar response to intraamniotic LPS, increasing markers of pulmonary inflammation and injury (Willet et al., 2002; Kallapur et al., 2011; Sosenko et al., 2006; Nadeau-Vallée et al., 2017). In contrast to these previous studies, repeated systemic LPS infusions did not alter lung development as evidenced by the lack of difference in tissue to airspace ratio, secondary septal crest density or elastin/collagen content within the parenchyma. The lack of a detectable inflammatory or structural response in LPS fetuses is likely due to three possibilities relating to the unique methodology employed in this study; a) the timing between LPS exposure and tissue collection; b) the lungs were not exposed to significant inflammation due to the fetal pulmonary circulation, as LPS was administered intravenously; or c) the lungs developed an endotoxin tolerance, due to increasing doses of LPS.

We measured the mRNA expression of several pro-inflammatory cytokines that are commonly upregulated during FIRS, including IL-1β and IL-6 (Kramer et al., 2010; Kelly et al., 2021; Gomez et al., 1998; Arntzen et al., 1998). Contrary to our hypothesis, IL-18 was the only pro-inflammatory cytokine that was upregulated. Fetal sheep were allowed a 2-day period of recovery between the last LPS bolus and post-mortem. In this 48-h period, it is possible that mRNA expression of pro-inflammatory cytokines returned to baseline levels within the lungs. Indeed, in a previous experiment performed by our group, plasma cytokine levels of IL-1β and IL-6 increased after each exposure to LPS but returned to baseline by day 3 of the experiment (Kelly et al., 2021). This is consistent with previous findings where an initial bolus of endotoxin caused an increase in markers of systemic inflammation, such as plasma cytokine levels of IL-6, IL-10 and TNF, which then returned to baseline by the end of the experiment, despite a progressive increase in endotoxin exposure, suggesting that the inflammatory response within the lungs is transient (Lear et al., 2014; Feng et al., 2009; Galinsky et al., 2020; Mathai et al., 2012). It is possible that during the 48 h period between the last bolus of LPS and post-mortem, the inflammatory cytokine mRNA levels in the lungs diminished, similarly to what was seen systemically (Liu et al., 2021). We did not collect lung tissue from earlier points in the experiment so are unable to confirm this contention. However, the lack of change in cellularity or CD45 expression indicates that this is unlikely as inflammatory cytokines recruit inflammatory cells, increasing the abundance of inflammatory cells in the lungs for several days (Kallapur et al., 2001). The lack of CD45 positive cells in animals who received LPS infusions suggests that this recruitment did not occur and therefore, it is unlikely that an inflammatory response occurred in the lungs.

The lack of response may be due to the unique fetal circulation. The lungs play a pivotal role in modulating FIRS in response to intrauterine inflammation. Moss et al. eloquently showed that amniotic fluid is required to come into contact with the fetal lungs to initiate a pulmonary inflammatory response in response to intraamniotic LPS exposure (Moss et al., 2002). However, in our study, LPS was administered systemically. During fetal development, the lungs are filled with liquid and play no role in gas exchange. Due to the presence of lung liquid, there is a high resistance to blood flow (pulmonary vascular resistance) and consequently, pulmonary blood flow is low. Indeed, the fetal sheep lungs receive ∼10% of right ventricular output at this stage of gestation, with the remaining 90% bypassing the lungs through the ductus arteriosus, back into the systemic circulation (via the descending aorta) (Rudolph, 1979). Further, fetal pulmonary blood flow has been shown to decrease after exposure to LPS (Galinsky et al., 2013b). Given that we administered LPS i.v to the fetus, the lungs may have not been exposed to a concentration of LPS sufficient to mount an inflammatory response. Instead, a higher dose of i.v LPS may be required to induce an inflammatory response within the fetal lungs compared to the brain. Indeed, surfactant proteins are particularly sensitive indicators of inflammation occurring within the lungs. As expression of surfactant protein mRNA in the LPS group was not different from controls, this does suggest that LPS did not reach the lungs in sufficient concentrations, as even a small amount of endotoxin can increase expression of surfactant proteins and expression can remain elevated for 15 days after endotoxin exposure (Bachurski et al., 2001). Taken together, these findings suggest that a fetal systemic inflammatory response may have little downstream consequence on pulmonary inflammation and injury.

Finally, the lack of an inflammatory response may be due to a tolerance to LPS developing in the fetal lungs. The low pulmonary blood flow likely exposed the lungs to a smaller dose of LPS than what was seen by other organs, including the brain (Kelly et al., 2021). This difference in dosage could have caused a tolerance to LPS to occur within the lungs which can reprogram the pulmonary innate immune response. LPS tolerance has only been examined in the context of intra-amniotic injection of LPS in fetal sheep. A dosage study by Kramer et al. found that 1–4 mg of intraamniotic LPS increased inflammation within the lung but at least 10 mg of LPS was needed to induce structural remodeling of the lung (Kramer et al., 2001). Interestingly, a study by Kallapur et al. administered 10 mg of LPS i.a to preterm fetal sheep 2 days or 2 and 7 days before delivery and found that the repeated exposure to LPS caused decreased expression of inflammatory cytokines *IL1B*, *IL6* and *IL8* in fetal sheep exposed to 2 doses of LPS compared to one (Kallapur et al., 2007). A limitation of the intra-amniotic method of administration is the inability to know how much LPS the fetus has been exposed to, due to the dependence on fetal breathing movements to consume LPS within the amniotic fluid. Similarly, due to low PBF in this model, we are not able to determine precisely how much LPS the fetal lungs were exposed to; thus, no conclusions can be drawn regarding the dosage of LPS and the extent of LPS tolerance induced.

LPS tolerance is associated with reduced TLR4 and CD14 signaling and reduced activation of NF-kB (Seeley and Ghosh, 2017; Nomura et al., 2000; Mengozzi et al., 1993). The disruption of these inflammatory pathways reduces the expression of pro-inflammatory cytokines that are typically secreted in response to LPS administration (Foster et al., 2007). The development of a tolerance to LPS would therefore account for the lower expression of *IL1B* and *IL6* mRNA in animals exposed to LPS, as activation of these cytokines occurs downstream of TLR4 signaling (Arntzen et al., 1998; Chen et al., 2017). A decline in TLR4 signaling and subsequent inflammatory pathways would prevent FIRS occurring within the fetal lungs. Consequently, fetal lung development is unaffected by systemic administration of LPS. It has been reported that 16–48 h is required for a robust endotoxin tolerance to develop in fetal sheep (Kallapur et al., 2007; Foster et al., 2007). This experimental timeline involved exposure to 3 LPS doses, administered 24 h apart, so it is possible that enough time had passed for the fetal sheep to develop endotoxin tolerance by the third exposure to LPS. Evidence of endotoxin tolerance exists within the analysis of our blood gas data. The increases in lactate, glucose and PaCO_2_ and decreases in pH, SaO_2_ and PaO_2_ that occur immediately after LPS administration are smaller after each bolus, despite the fact they are receiving double the dose each time. This trend is reflected in the blood pressure, heart rate and plasma cytokine responses following repeated LPS exposure (Kelly et al., 2021). This bi-phasic response suggests some form of systemic tolerance to LPS is occurring which may have been mirrored in the lungs. However, EEG power decreased in the LPS group compared to controls after each LPS bolus and did not return to baseline by the conclusion of the experiment. Additionally, analysis of the white matter tissue demonstrated that a profound inflammatory response and subsequent injury occurs following LPS exposure, suggesting that the brain did not initiate a tolerance response (Kelly et al., 2021).

Interestingly, we found that *IL18* mRNA expression was increased in LPS exposed lambs compared to controls, in contrast to the other genes investigated. IL-18 is a pro-inflammatory cytokine that helps to regulate the innate and adaptive immune responses (Ihim et al., 2022). While IL-18 shares many similarities to IL-1β, including similar signaling pathways, *IL18* expression has been shown to escape the effects of LPS tolerance (Zhu and Kanneganti, 2017). Verweyen et al. investigated mRNA expression of *IL1B*, *IL6* and *IL18 in vitro* after repeated exposure to LPS and found expression of *IL1B* and *IL6* were not different from control while *IL18* was significantly higher after repeated LPS exposure (Verweyen et al., 2020). These data demonstrate that LPS tolerance does not cause complete reprogramming of the innate immune system, as gene expression of certain pro-inflammatory cytokines are not always downregulated after repeated exposure to LPS. Although *IL1B, IL6 and IL18* expression is regulated by TLR signaling, *IL18* is unique in that its activation is also regulated by type I IFN signaling (Zhu and Kanneganti, 2017). While LPS tolerance reduces TLR signaling and therefore *IL1B* and *IL6* expression, the type I IFN signaling pathway remains functional and, as such, expression of IL18 is not affected by LPS tolerance (Zhu and Kanneganti, 2017).

Our findings are in contrast to Kramer et al. who, using a similar model of intravenous LPS to investigate the effect of FIRS on lung inflammation and remodeling, demonstrated increased expression of *IL6*, reduced alveolar wall thickness, abnormal distribution of elastin fibers and increased SP-B expression in the lungs of fetuses exposed to i.v LPS compared to controls (Kramer et al., 2009). The discrepancy in lung inflammation and structure between the two experiments could be attributed to differences in gestational age and the number of LPS doses used to provoke FIRS. Kramer et al. administered LPS to fetal sheep at a younger gestation (GA = 107 days compared to 129 days in our study) where the less developed lungs may have been more sensitive to endotoxins and unable to induce an LPS tolerance. It is possible that the developing immune system is more susceptible to disruptions to the balance of pro- and anti-inflammatory cytokines at younger gestations. Additionally, only one dose of LPS (100 ng) was administered (Kramer et al., 2009) compared to our 3 doses, albeit at much higher doses (300 ng, 600 ng, 1200 ng). Moreover, the study by Kramer et al. showed the extent of lung remodeling was higher at 7 days compared to 3 days after the LPS bolus (Kramer et al., 2009). As our study only allowed 2 days for lung maturation and remodeling to occur between the last LPS administration and post-mortem, it is possible that differences in lung structure would have been observed if more time was allowed for lung injury to establish. However, as 4 days had passed since the first LPS bolus, it is reasonable to have expected to see some degree of lung remodeling by the end of the experiment, similar to what was seen by Kramer et al. 3 days after LPS exposure. As such, it is probable that the differences between the two studies can be attributed to an endotoxin tolerance developing in the fetal lungs in our study, due to the higher number of LPS infusions used. Ultimately, we cannot conclude why the differences between the two studies occurred without further investigation into the development of endotoxin tolerance in preterm fetal lungs.

Contrary to our hypothesis, we found that the profound systemic inflammation resultant from intravenous LPS does not cause profound inflammation and remodeling in the fetal lungs. This is likely due to the experimental design, unique pulmonary circulation or to the development of fetal pulmonary tolerance. Taken together, our findings suggest that direct exposure to endotoxins within the lungs is necessary to produce a profound pulmonary inflammatory and structural response and that systemic born fetal inflammation may have limited effects on fetal lung development.

## Data Availability

The original contributions presented in the study are included in the article/supplementary material, further inquiries can be directed to the corresponding author.
